# Evaluation of plan quality and robustness of IMPT and helical IMRT for cervical cancer

**DOI:** 10.1186/s13014-020-1483-x

**Published:** 2020-02-13

**Authors:** Haijiao Shang, Yuehu Pu, Wei Wang, Zhitao Dai, Fu Jin

**Affiliations:** 1 Shanghai Institute of Applied Physics, Chinese Academy of Sciences, 201800 Shanghai, People’s Republic of China; 2 University of Chinese Academy of Sciences, 100049 Beijing, People’s Republic of China; 3 RaySearch China, 200120 Shanghai, People’s Republic of China; 4 Department of Radiation Oncology, Xinhua hospital affiliated to shanghai Jiao tong university school of medicine, Shanghai, People’s Republic of China; 5 National Cancer Center/National Clinical Research Center for Cancer/Cancer Hospital & Shenzhen Hospital, Chinese Academy of Medical Sciences and Peking Union Medical College, Shenzhen, People’s Republic of China; 6 School of Physics and Technology, Wuhan University, Wuhan, 430072 People’s Republic of China; 7 Department of Radiation Oncology, Chongqing University Cancer Hospital & Chongqing Cancer Institute & Chongqing Cancer Hospital, No. 181 Hanyu Road, Shapingba District, Chongqing, 400030 People’s Republic of China

**Keywords:** Intensity modulated proton therapy, Helical tomotherapy, Robust optimization, Normal tissue complication probability, Cervical cancer

## Abstract

**Background:**

Both plan quality and robustness were investigated through comparing some dosimetric metrics between intensity modulated proton therapy (IMPT) and helical tomotherapy based intensity modulated radiotherapy (IMRT) for cervical cancer.

**Methods:**

Both a spot-scanning robust (SRO) IMPT plan and a helical tomotherapy robust (TRO) IMRT plan were generated for each of 18 patients. In order to evaluate the quality of nominal plans without dose perturbations, planning scores (PS) on clinical target volume (CTV) and five organs at risk (OARs) based on clinical experience, and normal tissue complication probabilities (NTCP) of rectum and sigmoid were calculated based on Lyman-Kutcher-Burman (LKB) model. Dose volume histogram bands width (DVHBW) were calculated in 28 perturbed scenarios to evaluate plan robustness.

**Results:**

Compared with TRO, the average scores of SRO nominal plans were higher in target metrics [V_**46.8Gy**_, V_**50Gy**_, Conformity and Homogeneity](16.5 vs. 15.1), and in OARs metrics (60.9 vs. 53.3), including bladder [V_**35**_,V_**45**_, D_**mean**_,D_**2cc**_], rectum [V_**40**_,V_**45**_,D_**2cc**_,D_**max**_], bowel [V_**35**_,V_**40**_,V_**45**_, D_**max**_], sigmoid [V_**40**_,D_**max**_] and femoral heads [V_**30**_,D_**max**_]. Meanwhile, NTCP calculation showed that the toxicities of rectum and sigmoid in SRO were lower than those in TRO (rectum: 2.8% vs. 4.8%, *p* < 0.05; sigmoid: 5.2% vs. 5.7%, p < 0.05). DVHBW in target coverage for the SRO plan was smaller than that for the TRO plan (0.6% vs. 2.1%), which means that the SRO plan generated a more robust plan in target.

**Conclusion:**

Better CTV coverage and OAR Sparing were obtained in SRO nominal plan. Based on NTCP calculation, SRO was expected to allow a small reduction in rectal toxicity. Furthermore, SRO generated a more robust plan in CTV target coverage.

## Background

Cervical cancer accounts for almost 6.6 and 7.5% of female cancer morbidityand is the fourth leading cause of female cancer deaths [1]. As an advanced modality of radiotherapy for intensity modulated radiation therapy (IMRT) combined with an image guiding system, helical tomotherapy (HT) has been proven to be efficient for cervical cancer [2]. Due to the unique physical characteristic of Bragg peakand intensity modulated proton therapy (IMPT) would offer the best sparing healthy tissue as compared with IMRT, volumetric modulated arc therapy (VMAT), and HT, while maintaining excellent target coverage or conformity [3–5]. HT and IMPT share standard uncertainties in treatment delivery, including target definition, target motion, normal tissue motion, and patient setup uncertainties, which sets the margin from the clinical target volume (CTV) to planning target volume (PTV) based on clinical experience in the process of the radiotherapy [6]. Studies in prostate cases have also shown that PTV-based IMPT has comparable target coverage and reduces rectal toxicity to HT [7].

The PTV concept, as typically applied in IMRT planning, relies on the assumption that the dose distribution in the treatment room is not affected by changing in the patient’s anatomy. That is, CTV is expected to receive a prescribed dose as long as it stays within the PTV. However, this fundamental assumption does not always works, especially for IMPT, in which anatomical misalignment can lead to significant dose distortion at the edges of the PTV and even inside the planning target [8, 9]. Recently, a novel strategy to deal with the uncertainties is to develop a robust optimization algorithm instead of using PTV-based optimization, which has been reported to be effective in compensating for setup and range uncertainties in both proton and photon radiotherapy [10–14].

The present paper directly compares CTV-based IMPT and HT plans, in which uncertainties caused by patient setup and CT density are taken into account. To our knowledge, this is the first study to compare plans with different beam modalities using the robust optimization method. We perform a specific comparison between them in terms of plan quality and robustness for cervical cancer. Furthermore, several novel tools for quantitative analyzing plan quality and robustness are developed.

## Materials and methods

### Patients selection and contouring

A retrospective study including 18 patients with cervical cancer who had undergone postoperative radiotherapy was carried out and approved by the local ethics committee. According to the International Federation of Gynecology and Obstetrics, these patients were classified as stage IIB and III (A and B) and are usually treated with combined chemotherapy and radiotherapy [15]. Gross target volume (GTV) and OARs were defined according to International Commission on Radiation Units & Measurement Report 50 and 83, in which prescribing, recording, and reporting doses have also been standardized [16, 17]. CTV was separated into primary (pCTV) and nodal (nCTV) components according to consensus guidelines for delineation [18]. pCTV includes the GTV, cervix, uterus, parametria, ovaries, and vaginal tissues, and nCTV includes involved nodes and relevant draining nodal groups. OARs, including bone marrow, femoral heads, bladder, rectum, spinal cord, sigmoid, and small bowel, were delineated.

### Treatment planning

Plans based on robust optimization method for both HT and IMPT were created for all patients using the RayStation treatment planning system (RaySearch Labs, Version 8B, Sweden). The robust optimization method in RayStation is based on the min-max optimization [19], in which it is planned to optimize in multiple geometries, and the worst (maximum) objective value from these geometries is used in the objective function. For the plan with tomotherapy robust optimization (TRO), a field width of 2.5 cm, the pitch of 0.287, and modulation factor of 3.0 to 3.5 are produced. The resulting irradiation time is typically in the range of 8 to 10 min. The isocenter offsets are applied in the specified direction, which defines the volume for which the plan would be robust. The shifted values from the isocenter are 5 mm in the anterior-posterior, left-right, and superior-inferior directions.

The plans to implement IMPT with spot-scanning robust optimization (SRO) were created using a proton beam therapy system called Shanghai Advanced Proton Therapy (SAPT; Shanghai Institute of Applied Physics, China). Ninety-four energy bins between 70 and 235 MeV were available for SAPT facility [20]. The full width at half maximum (FWHM) of the spot size in air at the isocenter varied from 4 mm (at 230.0 MeV) to 6 mm (at 70 MeV), and the ellipticity of the beam spots was close to zero. The spot spacings in both the horizontal and vertical directions were determined automatically and ranged from 4.8 to 5.6 mm in this study. There are several beam angles to choose from for the treatment of the whole pelvic region in proton therapy. Lin et al. used the posterior oblique field technique [21], and Marnitz et al. used the three-field technique [3]. In current study, left and right parallel fields were applied to avoid beam range uncertainties. In this study, the dosage unit of Gray (Gy) represents a dose weighted by the relative biological effectiveness (RBE), and RBE value of 1.0 and 1.1 were employed for the TRO and SRO plan, respectively. The prescribed dose was 46.8 Gy in 26 fractions. Dosimetric constraints for target volume were as following: 95% of the CTV received the prescription dose, at least 99% of the CTV received 90% of the prescribed dose, and no more than 5% of the CTV received 107% of the prescribed dose.

### Robust optimization criteria

Both TRO and SRO plans were 3D CTV–based robustly optimized, accounting for several scenarios in which patient setup and range uncertainties (only for SRO) were simulated. Setup uncertainty was stimulated by shifting the plan isocenter and range uncertainty by scaling the planning CT density. A uniform 5-mm patient setup and ± 3.5% range uncertainties were considered for robust objective functions according to Harald’s studies [22]. Two additional manual structures x-mm rings were used to ensure the dose conformability, while the dose fall-off function for the external dose was also used to limit low-dose spillage. Generalized equivalent uniform dose (gEUD) objective functions [23], which related to biological effect, were also used for all OARs. The details of optimization objectives are shown in Table 1.

**Table 1:** Cost functions used in robust optimization

Region of interest	Dose Objective	Weight	Robust
CTV	Minimum dose 46.8 Gy	70	On
	Uniform dose 47 Gy	80	On
	Maximum dose 48G y	50	On
External	Dose fall-off: high dose 46.8 Gy, low dose 20 Gy, 10-mm distance	10	
3-mm ring	Max EUD 45 Gy, A = 150	5	
10-mm ring	Max EUD 20 Gy, A = 150	5	
Bladder	Max Dose 48 Gy	10	
	Max EUD 30 Gy, A = 2	5	On
Rectum	Max Dose 48 Gy	10	On
	Max EUD 30 Gy, A = 2	5	
Spinal cord	Max Dose 10 Gy, A	2	
Bowel	Max Dose 48 Gy	15	
	Max EUD 30 Gy, A = 2	5	
Sigmoid	Max Dose 48 Gy	15	
	Max DVH 40 Gy, 20%	10	
Femoral heads	Max EUD 15 Gy, A = 2	2	

### Evaluation

#### Quick plan review

A plan score (PS) template was developed to quickly perform a comparison of the plans, in which a series of indices and DVH metrics were listed for all formal ROIs. The total score points may be described by the following formula 1:

1 $$ \mathrm{SD}=\sum \limits_{j=1}^K{S}_j $$

where *K* is the total number of metrics, SD is the total scoring point, and *S*_*j*_ is the individual scoring point for each metric.

The scoring metrics were divided into two groups: one is the target, and the other is the OAR. The target (2) and OAR scoring formula (3) can be written as following:

2 $$ {S}_T=\left\{\begin{array}{c}0\ M\le X1,\\ {}\frac{\left|M-X1\right|}{\left|X2-X1\right|}\times P\ X1\le M\le X2\\ {}P\ M\ge X2\end{array}\right., $$

3 $$ {S}_O=\left\{\begin{array}{c}0\ M\ge X2,\\ {}\frac{\left|M-X2\right|}{\left|X2-X1\right|}\times P\ X1\le M\le X2\\ {}P\ M\le X1\end{array}\right., $$

where *M* is the actual value of target, *X*1 and *X*2 are the worst and best value, respectively, and *P* is the scaling score points with each target metric.

The PS system consists of 20 metrics (*K* = 20), which are based on local physician clinical experience. The total scores are assigned 100 points, with the specific allocation is as follows: *S*_*T*_ metrics [1–4] with total points of 24 are used to calculate the points of CTV dose coverage [V46.8Gy (%), V50 Gy (%)], conformity number (CN) and homogeneity index (HI). The rest 76 points are *S*_*O*_ metrics [5–20] are assigned to specific dose-volume parameters of five OARs, where rectum, bowel, bladder, sigmoid and femoral heads shared 23, 22, 15, 9 and 7 points, respectively. The details of score points are shown in Table 2. The PS metrics are developed based on Cancer Hospital Chinese Academy of Medical Sciences in Shenzhen (CAMS) clinical requirement. In practice, we refer to the PS metrics covering a broad range [from Worst (X1) to Best (X2)] to meet clinical goals for all patients.

**Table 2:** Details of dosimetric criteria in the planning score (PS) system

Evaluation metric		Worst(X1)	Best (X2)	Score(X1)	Score(X2)
CTV	V_46.8Gy_ (%)	< 90%	≥98%	0	10
	V_50Gy_ (%)	≥10%	≤0%	0	6
	CI	≤0.6	≥0.95	0	4
	HI	≥0.2	≤0.0	0	4
Bowel	D_max_ < 50Gy	≥50Gy	≤48Gy	0	8
	V_45Gy_ < 65 cc	≥80	≤40	0	5
	V_40Gy_ < 100 cc	≥140	≤90	0	8
	V_35Gy_ < 180 cc	≥220	≤150	0	3
Rectum	V_40Gy_ (%) < 50%	≥60%	≤30%	0	4
	V_45Gy_ (%) < 30%	≥45%	≤20%	0	5
	D_2cc_ < 49.5 Gy	≥50Gy	≤45Gy	0	6
	D_max_ < 50Gy	≥50Gy	≤46Gy	0	6
Sigmoid	D_max_ < 50 Gy	≥50Gy	≤48Gy	0	5
	V_40Gy_ < 100 cc	≥120	≤80	0	4
Bladder	D_mean_ < 30 Gy	≥35Gy	≤20Gy	0	5
	V_45Gy_ (%) < 40%	≥60%	≤30%	0	3
	V_35Gy_ (%) < 50%	≥80%	≤40%	0	3
	D_2cc_ < 49.5 Gy	≥50Gy	≤48Gy	0	4
Femoral heads	V_30Gy_ (%) < 15%	≥20%	≤10%	0	4
	D_max_ < 45 Gy	≥50Gy	≤40Gy	0	3

CTV dose coverage is described as when 95% of the target volume (CTV) received a minimum of 100% of the prescription dose (CTV :*V*_46.8 Gy_ ≥ 95%) and at most 5% of the target volume (CTV) received a maximum of 107% of the prescription dose (CTV :*V*_50 Gy_ ≤ 5%).

The Conformity Number CN (4) was defined as follows:

4 $$ \mathrm{CN}=\frac{V_{t, ref}}{V_t}\times \frac{V_{t, ref}}{V_{ref}} $$

Where, ***V***_***t*,*ref***_ means the volume of target covered by prescription dose, ***V***_***t***_ means volume of target; ***V***_***ref***_ means volume covered by prescription dose.

The Homogeneity index HI (5) can be was defined as follows:

5 $$ \mathrm{HI}=\frac{D_{2\%}-{D}_{98\%}}{D_{50\%}} $$

Where D_2%_, D_50%_, D_98%_ means the dose received by 2, 50, 98% volume of CTV, respectively.

According to the PS assessment, the plan with a higher point score indicates better plan quality. Average score points are compared between TRO and SRO plans.

#### Normal tissue complication probability (NTCP)

As the most dominant OAR in cervical radiotherapy, the dose range of rectum has much clinical significance. For instance, studies show that rectum bleeding is associated with rectum high dose range [ 24]. The popular Lyman-Kutcher-Burman (LKB) NTCP model was used to fit the dose volume relationship to the clinical data [25, 26]. However, there are significant uncertainties in the NTCP model and its associated parameters, which might result in the scoring complications, disparity in endpoints, and dosimetric changes. In current studies, biology models have described the steep dose-response relationships established for rectum and sigmoid from large groups of gynecological cancer survivors for a 2- to 14-year follow-up [27]. In Eleftheria’s study [27], large groups and long history follow-up in gynecological cancer, make the model parameters best suited for their data, with parameters of the LKB model are show from Table 3. In our study, the LKB model for NTCP calculation was performed for rectum and sigmoid in the TRO and SRO plans, respectively. A Student *t*-test was performed to compare the pairwise difference between the TRO and SRO plans for sigmoid and rectum using the LKB model. A *p*-value < 0.05 was considered statistically significant.

**Table 3:** NTCP parameters used in the Lyman-Kutcher-Burman model

Organ	D50 Gy]	M	N	α/β [Gy]
Rectum	51.5	0.47	9 ' 10^6^	0.63
Sigmoid	51.3	0.44	0.079	1.18

#### Plan robustness

Plan robustness can be evaluated by calculating the perturbed dose through shifting plan isocenter with 5 mm and scaling CT density with ±2% uncertainties according to previous the study [22]. All perturbed doses were calculated under two formation with 28 scenarios: one creation is 12 with ±5 mm in the Axes endpoints directions under ±2% CT density shifts, and the other is 16 with ±5 mm in Diagonal endpoints directions under ±2% CT density shifts. The detail of the scenarios is shown in Table 4.

**Table 4:** Patient position uncertainty: two formations with 28 scenarios

Axes Endpoints	One creation with 12 scenarios											
	1	2	3	4	5	6	7	8	9	10	11	12
X (mm)	5	−5	0	0	0	0	5	−5	0	0	0	0
Y (mm)	0	0	5	−5	0	0	0	0	5	−5	0	0
Z (mm)	0	0	0	0	5	−5	0	0	0	0	5	−5
CT density	2%	2%	2%	2%	2%	2%	−2%	− 2%	− 2%	− 2%	− 2%	− 2%
Diagonal Endpoints	The other creation with 16 scenarios											
	1	2	3	4	5	6	7	8	9	10	11	12
XY (mm)	2.9	2.9	−2.9	− 2.9	− 2.9	2.9	2.9	− 2.9	2.9	2.9	−2.9	− 2.9
YZ (mm)	2.9	2.9	2.9	2.9	−2.9	− 2.9	− 2.9	− 2.9	2.9	2.9	2.9	2.9
XZ (mm)	2.9	−2.9	2.9	−2.9	− 2.9	− 2.9	2.9	2.9	2.9	−2.9	2.9	−2.9
CT density (%)	2%	2%	2%	2%	2%	2%	2%	2%	−2%	− 2%	− 2%	− 2%
	**13**	**14**	**15**	**16**								
XY (mm)	−2.9	2.9	2.9	−2.9								
YZ (mm)	−2.9	−2.9	−2.9	− 2.9								
XZ (mm)	−2.9	−2.9	2.9	2.9								
CT density (%)	−2%	−2%	−2%	−2%								

*X* right-Left, *Y* Inferior-Superior, *Z* posterior-anterior

DVH band width (DVHBW) was calculated on the same coordinate axis, displaying the DVH plot for all perturbed doses. The DVH metric of D_95%_ for CTV was selected, and DVHBW definition can be seen in formula (6) as follows:

6 $$ \Big\{{\displaystyle \begin{array}{c}\varDelta D={D_{95\%}}^{s_{max}}-{D_{95\%}}^{s_{min}}\\ {}\uplambda =\mid \frac{\varDelta D}{D_P}\mid \times 100\%\end{array}} $$

where ${D_{95\%}}^{s_{max}}$ and ${D_{95\%}}^{s_{min}}$ represent the best and worst target coverage in certain scenarios, respectively. Therefore, *ΔD* indicates the largest dose difference over the range of all uncertainties. λ is a relative value that DVHBW was scaled by the prescription dose D_P_. λ was used to quantify the plan robustness.

Clinical goals in the worst scenarios were considered using either the voxel-wise minimum or maximum goals [28]. For clinical goals in targets with the least value requirements, the minimum voxel-wise distribution will be used. For clinical goals in OARs with at-most value requirements, the maximum voxel-wise distribution will be used. The DVH metrics in the CTV [D_**95%**_, D_**50%**_] are counted based on voxel-wise min distribution, and the DVH metrics in rectum [D_**2cc**,_ D_**max**_] are also computed based on voxel-wise max distribution. A *p*-value < 0.05 was considered statistically significant.

## Results

### Plan quality

**Fig. 1:**
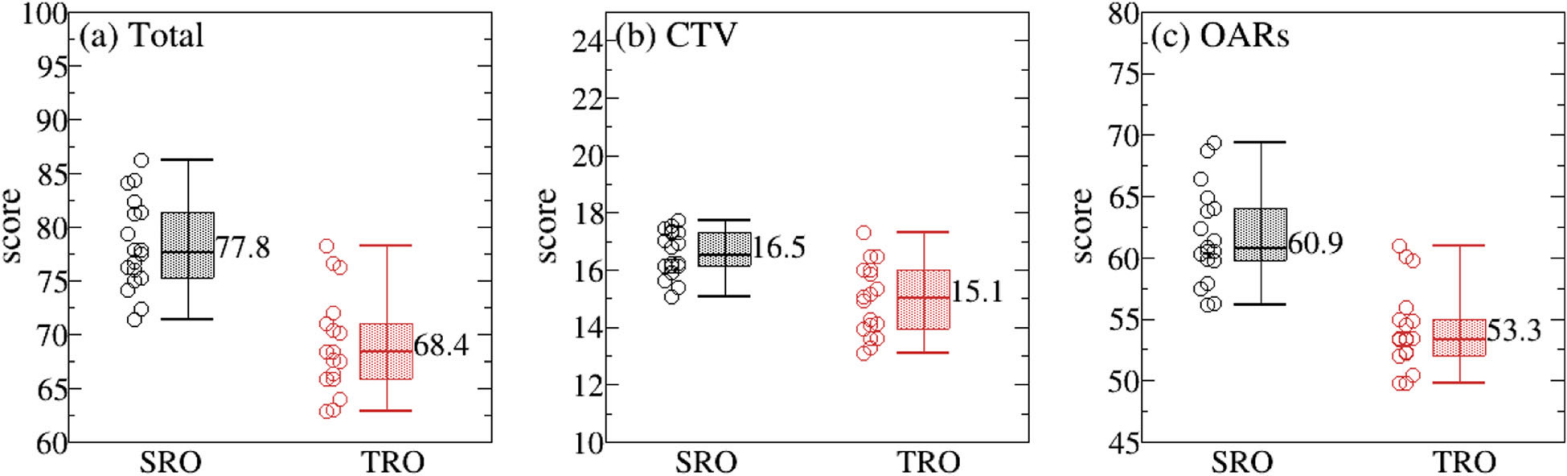
Comparison of SRO and TRO plan in total plan score points (**a**), target score points (**b**), and OAR score points (**c**)

The previous studies [3, 7] indicated that IMPT resulted in lower doses in rectum and bladder than HT for pelvic cancer. In our studies, the robust optimization method was introduced to compare the IMPT (spot scanning) and HT treatment plan for patients with cervical cancer. Figure 1a-c shows the results obtained using PS.

**Fig. 2:**
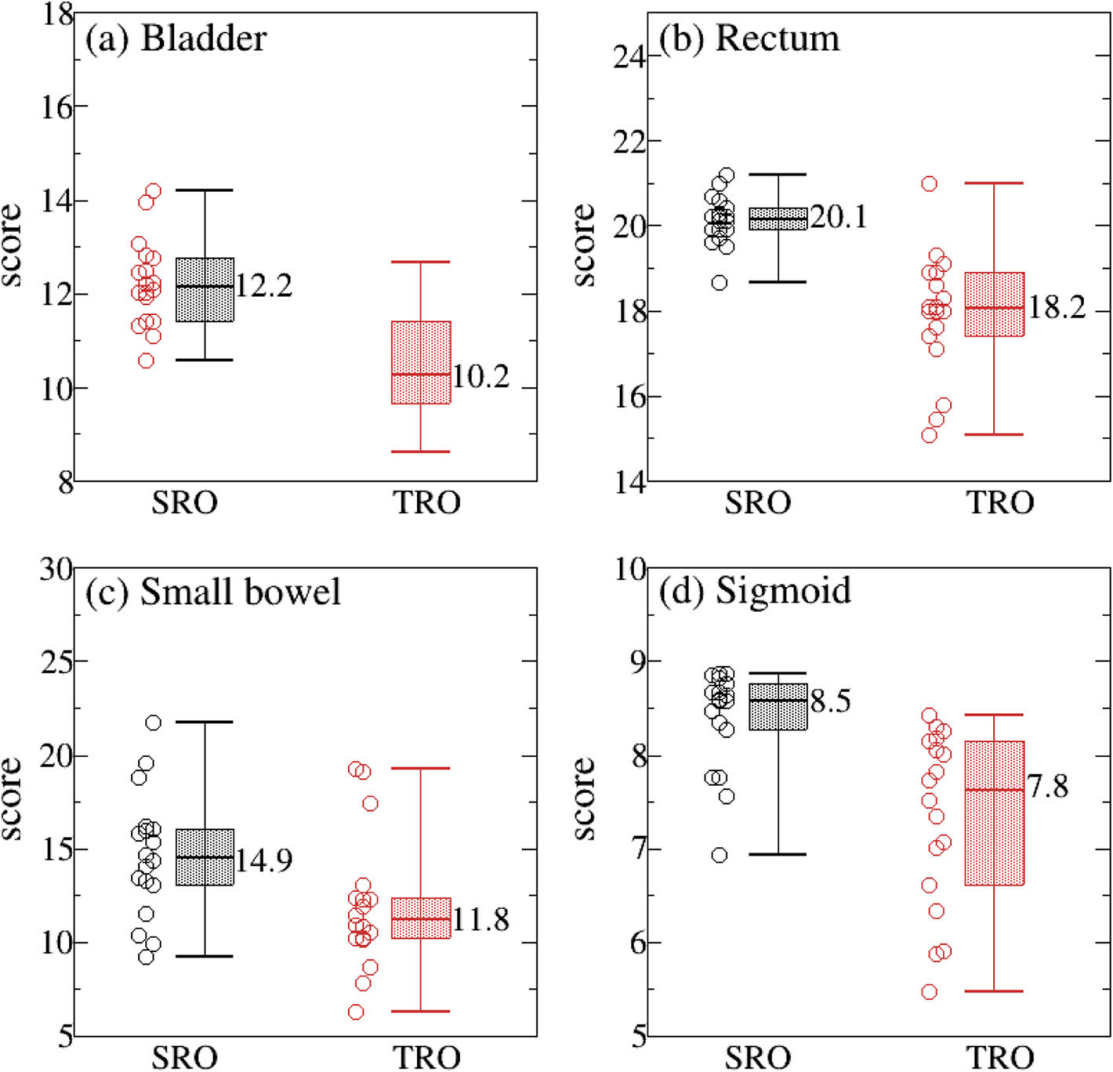
Comparison of SRO and TRO plan in bladder score points (**a**), rectum score points (**b**), small bowel score points (**c**), and sigmoid score points (**d**)

**Fig. 3:**
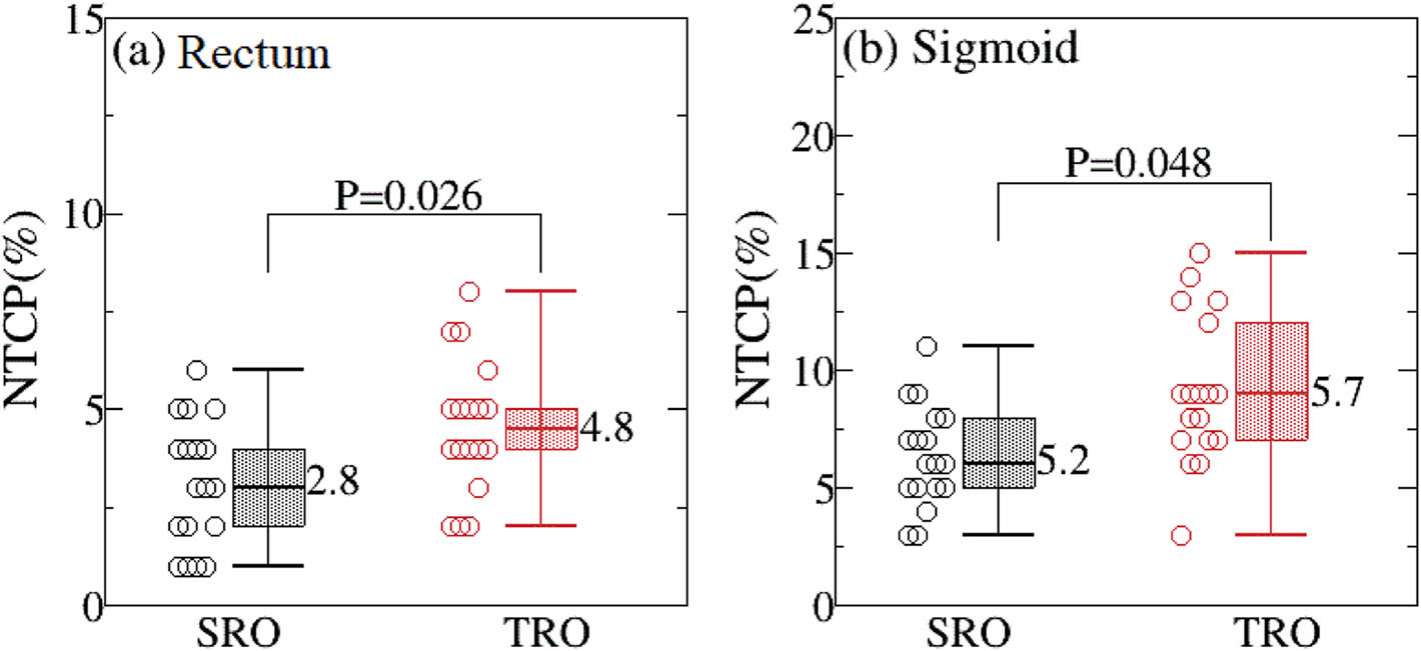
Comparison of NTCP values for SRO and TRO plan in rectum (**a**) and sigmoid (**b**); all values showed a significant difference (*p* < 0.05). The *p*-value was determined using a Student *t*-test

As can be seen from Fig. 1, the average total score points for SRO were significantly higher than that of TRO plans (77.8 for SRO and 68.4 for TRO), which is not only reflected in the target coverage (16.5 for SRO and 15.1 for TRO), but also in the OARs (60.9 for SRO and 53.3 for TRO). Looking at the OARs in more detail, bladder, rectum, sigmoid, and small bowel were all observed to have higher points in the SRO than in the TRO plan, respectively (bladder: 12.2 for SRO and 10.2 for TRO; rectum: 20.1 for SRO and 18.2 for TRO; small bowel: 14.9 for SRO and 11.8 for TRO; sigmoid: 8.5 for SRO and 7.8 for TRO; Fig. 2a-d). The difference was obvious for all OARs, which is consistent with results obtained in prostate studies [7]. Considering the NTCP, rectum and sigmoid calculated using the LKB model were compared in both SRO and TRO plans. As were displayed in Fig. 3, NTCP of rectum and sigmoid were significantly lower in the SRO plans (rectum = 2.8% for SRO and 4.8% for TRO; sigmoid = 5.2% for SRO and 5.7% for TRO; *p* < 0.05).

### Plan robustness

**Fig. 4:**
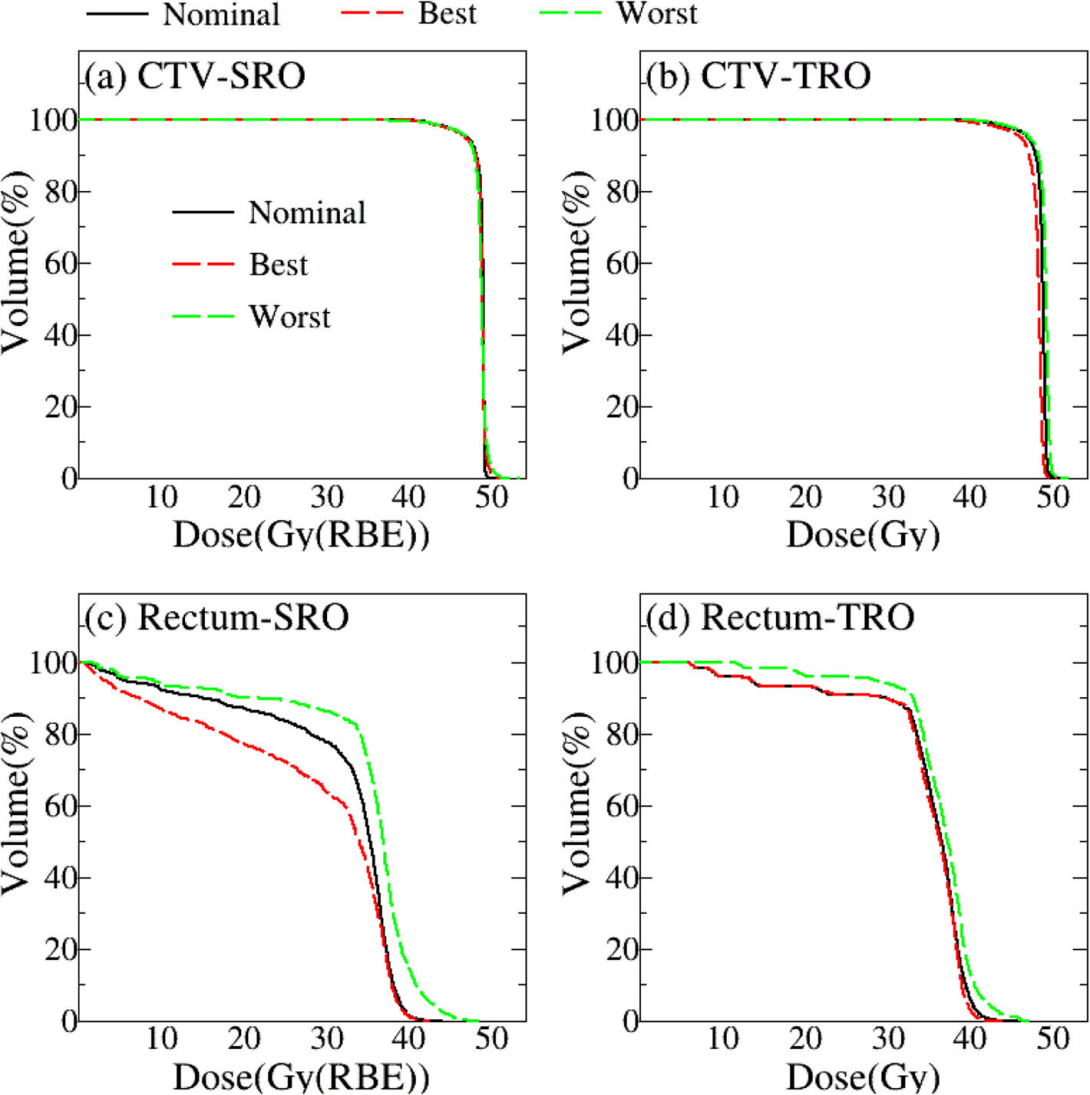
Dose volume histogram showing the dose to the target volumes and rectum for SRO (**a**, **c**) and TRO (**b**, **d**). The black solid line represents the nominal plan DVH, green dot line represents the worst DVH and red dot line represents the best DVH

**Fig. 5:**
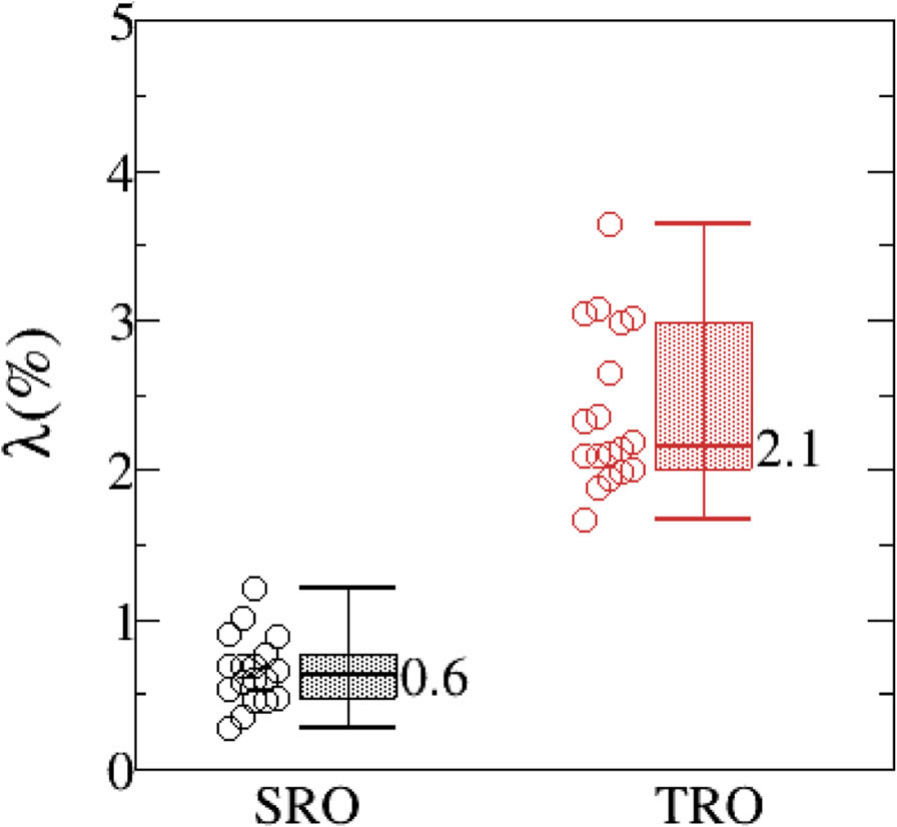
Comparison of λfor SRO and TRO plans

Previous studies have proven that robust optimization is effective for plan robustness in photon and proton treatment planning [10, 13]. In our research, plan robustness was evaluated in both proton and photon plans with robust optimization. Figure 4 shows the results obtained using a representative case. As can be seen from Fig. 4a, b, the DVH bands of CTV in SRO plan look narrower than that in TRO plan, but no evidence showed that occurred in rectum Fig. 4c, d. A quantitative analysis to determine plan robustness was applied statistically, and it was observed that λfor SRO plans was significantly smaller than that for TRO plans (λ = 0.6 % for SRO and 2.1 % for TRO), as shown in Fig. 5.

**Fig. 6:**
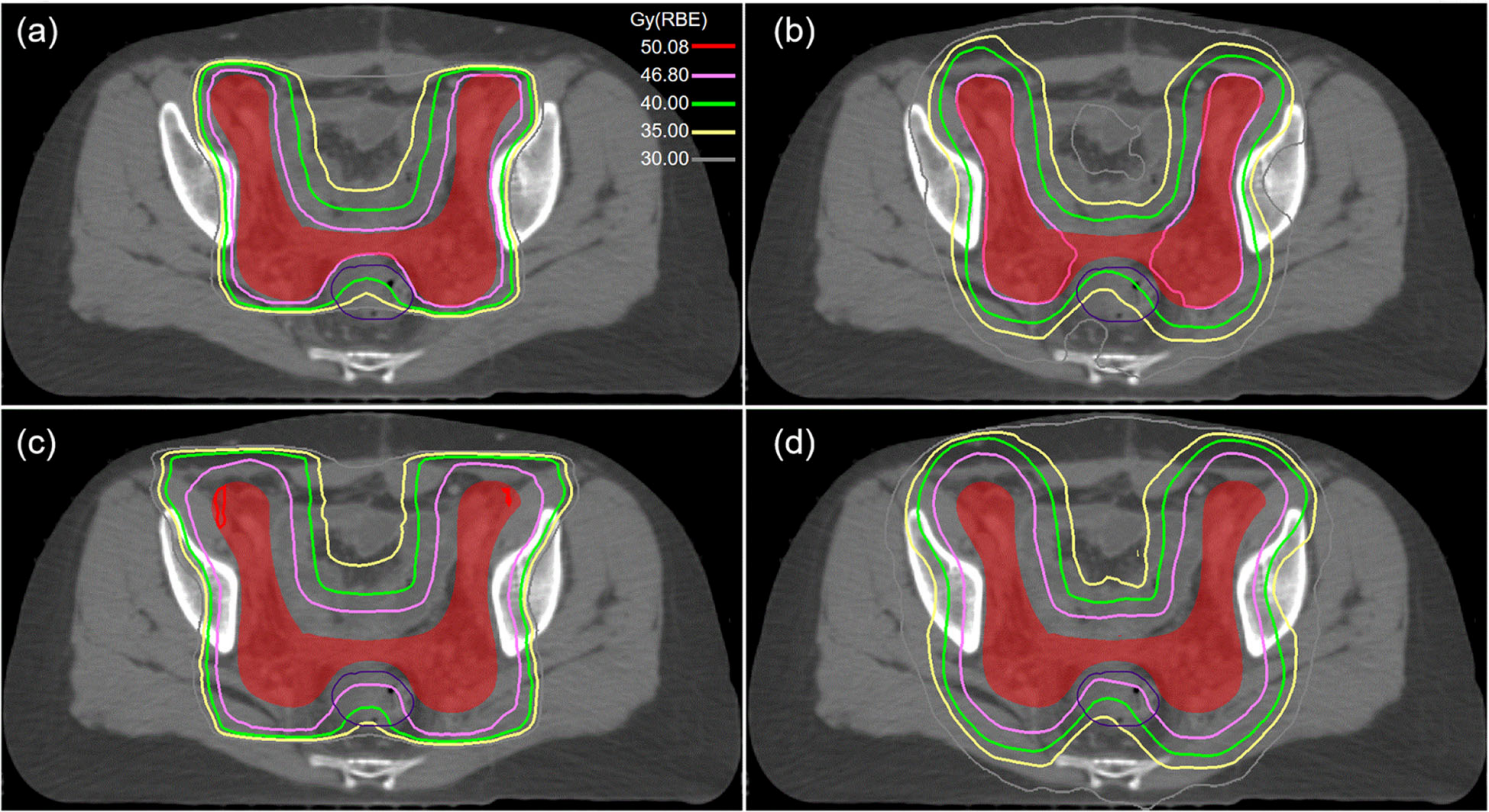
Voxel-wise minimum dose distribution and voxel-wise maximum dose distribution in the transverse plane for a selected case: (**a**) minimum dose distribution of SRO plan; (**a**) minimum dose distribution of TRO plan; (**C**) maximum dose distribution of SRO plan; (**a**) maximum dose distribution of TRO plan

Voxel-wise dose distribution is another metric for evaluating plan robustness. Figure 6 shows the voxel-wise minimum dose distribution and voxel-wise maximum dose distribution in the transverse plane for a selected case. As can be seen from Fig. 6a and b, the prescription dose line covers more CTV volume in the voxel-wise minimum dose distribution in the SRO plan. However, the low dose range (for instance 30 Gy) covers similar rectum volume from the voxel-wise max dose distribution, as shown in Fig. 6c and d. The results demonstrate that the proton plan using the robust optimization method maintain target coverage but may not spare OAR dose under worst scenario.

DVH metrics in representative ROIs (CTV, rectum) were also analyzed in the voxel-wise dose distributions, where CTV coverage and rectum metrics were exacted in voxel-wise minimum and maximum dose distribution, respectively. As were detailed in Table 5, CTV coverage for SRO plans was significantly better than that for TRO plans. DVH metrics in CTV [D_95%;_ D_50%_] for SRO were statistically different (D_95% Gy_: [45.9 vs. 45.5, *p* = 0.002]; D_50%_: [47.9 vs. 47.4, *p* = 0.046]). But there is no significant difference for the maximum dose [D_max Gy_ and D_2cc Gy_] of rectum between SRO and TRO plans. These results indicate that the SRO plan is more robust for CTV but not for OAR, which is consistent with Fig. 4c, d.

**Table 5:** Dose-volume parameters of the SRO and TRO plans in the worst scenarios

Structure	SRO		TRO		*p*
	Median (Gy)	Range (Gy)	Median (Gy)	Range (Gy)	
CTV					
D_95%_	45.9	(45.5–46.5)	45.5	(45.3–46.3)	0.002
D_50%_	47.9	(47.4–47.9)	47.4	(47.2–48.3)	0.046
Rectum					
D2cc	50.7	(48.4–50.8)	50.8	(48.5–51.2)	0.57
D_max_	51.3	(49.8–51.4)	51.3	(49.8–51.8)	0.32

## Discussion

Prior work has documented the advantage of proton therapy in sparing OARs compared with photon therapy while maintaining excellent target coverage. Marnitz [3] reported that proton offered the best sparing of small bowel and rectum, which lead to reduction in toxicity in cervical cancer treatment. However, that study was performed based on the PTV concept that has been quite controversial in both photon and proton radiotherapy [9, 10]. As a solution based on CTV, robust optimization has been proven to protect normal tissue in various tumor sites [10–14]. In our study, the robust optimization technique was introduced for both the SRO and TRO plans for cervical cancer, and plan quality and robustness were investigated.

The same patient setup uncertainties were applied in robust optimization for both SRO and TRO. However, range uncertainties due to electron density conversion should be taken into account in SRO plan, which is negligible for photon planning. This makes the objective function hardly available in the process of SRO. Despite this, it is found that SRO plans still provided superior plan quality and better robustness than TRO plans. These findings are consistent with the previous studies that proton could significantly reduce toxicities of OARs in cervical cancer treatment. Besides, two novel tools for quantitative analysis, PS and DVHBW, were developed to evaluate plan quality and target coverage robustness in both PRO and TRO plans. It is indicated these tools may enable us to assess the plan quality and robustness more efficiently and quantitatively.

While compared with traditional DVH metric methods, the in-house PS is a convenient way for quantitatively evaluating the plan quality, since a higher score indicates better plan quality. Our results show that the total score of the plan is more elevated in SRO plans than in TRO plans (77.8 vs. 68.4), which is mainly attributed to the OARs score points (60.9 vs. 53.3). From the score with each OAR in more detail, many differences were found for small bowel (14.9 vs. 11.8), balder (12.2 vs. 10.2), and rectum (20.1 vs. 18.2). When focusing on dosimetric criteria (as shown in Table 2), lower maximum doses were obtained for all the OARs in PRO plan.

Although randomized clinical trials are necessary to confirm whether proton therapy reduces toxicity as compared with photon therapy, it is hard to perform such studies. NTCP modeling analysis is one method to estimate the effectiveness of proton over photon radiotherapy from previous studies [29–31]. NTCP of rectum and sigmoid were calculated based on LKB model. The model parameters were taken from Eleftheria’s study [27] that enrolled a database of 2- to 14-year follow-up. The present study shows that NTCP values for rectum and sigmoid in the proton plan are significantly lower in SRO plans, which is consistent with previous studies [ref].

DVHBW in target coverage was used to simplify the comparison of plan robustness for SRO and TRO plans, which allows us to evaluate plan robustness with a single value quickly. It was found that the average target DVHBW in the SRO plan was significantly smaller than that in the TRO plan (0.6% vs. 2.1%, *p* < 0.05). We might conclude that robust optimization methods are more useful for proton therapy in target. Also, Voxel-wise minimum/maximum distribution in the worst scenario as a supplementary method to DVHBW could take into consideration other DVH metrics for ROIs, such as target and rectum. The DVH metrics show that SRO plans provide better target coverage under the worst scenario, but the maximum dose of rectum might not gain the advantage from robust optimization method.

Most notably, this is the first study to our knowledge to compare plan differences concerning plan quality and plan robustness between two beam modalities. Our results provide compelling evidence for IMPT planning with robust optimization, which shows excellent promise for sparing OARs, especially for bladder, rectum, bowel, and sigmoid. However, some limitations are worth noting. Several factors are known to affect radiotherapy dose distribution in the treatment of cervical cancer. The pelvic organs at risk inherently tend to show positional and anatomical variation over time. Any variation in bladder and rectum filling can cause change in target position and shape. Traditional robust optimization or evaluation is not sufficient to account for the positional and anatomical variation in both target and OARs. Considerable effort is needed for developing better robust optimization algorithms to handle these uncertainties [32]. If this is realized, of advantage IMPT might be made full use in sparing healthy tissue while maintaining target coverage in scenarios of uncertainties.

## Conclusion

Previously studies show that robust optimization is useful to improve the plan quality and robustness for photon and proton. In current studies, both plan quality and robustness were investigated by comparing SRO and TRO for cervical cancer. The results showed that better CTV coverage and OARs sparing were observed in SRO nominal plan. Based on NTCP calculation, SRO was expected to allow a small reduction in rectal toxicity. Furthermore, SRO generated a more robust plan in CTV target coverage.

## Data Availability

All data included in this study are available upon request by contact with the corresponding author.

## Abbreviations

CTV: Clinical target volume
DVHB: Dose-volume histogram bands
gEUD: Generalized equivalent uniform dose
NTCP: Normal tissue complication probability
RTOG: Radiation therapy oncology group
SRO: spot-Scanning robust optimization
TRO: Tomotherapy robust optimization
